# Decoding of naturalistic textures from spike patterns of neuromorphic artificial mechanoreceptors

**DOI:** 10.1186/1471-2202-16-S1-P186

**Published:** 2015-12-18

**Authors:** Alberto Mazzoni, Udaya B Rongala, Calogero M Oddo

**Affiliations:** 1 The BioRobotics Institute, Scuola Superiore Sant'Anna, Viale Rinaldo Piaggio 34, Pontedera 56025, Pisa, Italy

## 

Significant advances were recently achieved in the bidirectional control of upper limb neuroprostheses [1], however implementing a realistic tactile feedback on artificial hands is still an open challenge [2]. We target this ambition with a neuromorphic approach. Towards this direction we integrated MEMS tactile sensors with a realistic spatial arrangement on the distal phalanx of artificial fingers [3] and we converted the sensor readouts into spike trains mimicking the neural firing properties of glabrous skin mechanoreceptors [4]. The neuromorphic approach presents several advantages [5], among which the most relevant for neuroprosthetics is that a sufficiently realistic tactile feedback is expected to reduce drastically the time the brain needs to adapt to the prosthesis and to each single new task, leading to enhanced quality of life of the subjects.

Since the long term goal is to implement a hardware version of the neuromorphic neurons, we modeled the mechanoreceptors as Izhikevich neurons, a model which at the same time is computationally lean and takes into account the effects of firing rate adaptation that are particularly relevant in mechanoreceptors. Parameters were tuned so to achieve a match with Slowly Adapting (SA) mechanoreceptors dynamics in primates [4]. The output of each tactile sensor was normalized and injected as an excitatory input current into a single neuron model [6]. To reproduce the features of Fast Adapting (FA) mechanoreceptors responsible for edge detection [7], we injected in a second set of neuron models the smoothed derivative of the pressure sensors outputs.

The fingertip was then repeatedly presented with ten different daily-life textures such as textiles, polymeric tissues, glass or paper. FA spike trains were used to identify onset and offset of contact between fingertip and stimuli, whereas SA spike trains were used to decode the stimulus presented. We applied two decoding procedures: one based on the combination of firing rate and inter-spike interval Fano factor, and the other one based on Victor-Purpura distance [8]. Error rate was close to 20% in the first case and to 5% in the second, reflecting the relevance for decoding of the fine temporal structure of the neuromorphic sensor outputs. Finally, we introduced a normalization in the cost of the Victor-Purpura spike train metrics thanks to which the above level of correct classification was maintained even if sensing conditions varied across trials. These results support a possible future evaluation of the biomimetic fingertip endowed with neuromorphic artificial mechanoreceptors in clinical trials with human subjects.
